# A declaration for all?

**DOI:** 10.4102/phcfm.v11i1.2005

**Published:** 2019-05-21

**Authors:** Ian Couper

**Affiliations:** 1 Ukwanda Centre for Rural Health, Faculty of Medicine and Health Sciences, Stellenbosch University, Cape Town, South Africa

The year 2018 marked the 40th anniversary of the Alma Ata Declaration on Primary Health Care (PHC), adopted at Almaty, Kazakhstan, in September 1978. Primary Health Care comprises of three aspects that focus on improving the health of individuals and communities, namely, empowering people, multisectoral policy and action, and integrated health services built around primary care and public health. Many different events took place during 2018 in preparation for a Global Conference on Primary Health Care, held on 25–26 October 2018, arranged by the World Health Organization (WHO) and UNICEF (United Nations Children’s Fund) in Astana, Kazakhstan, where various world governments renewed their commitment to PHC in order to achieve universal health coverage and the 2030 Sustainable Development Goals.

In the lead-up to this event, the 15th World Rural Health Conference took place in Delhi, India, in April 2018, organised under the auspices of Rural Wonca, the rural working party of the World Organisation of Family Doctors (WONCA). This conference was held in the full knowledge of the plans to celebrate the Alma Ata anniversary. A National Consultation on Strengthening Primary Health Care in Rural India was nested within the conference, hosted by the Academy of Family Physicians of India. The report of this consultation, available at http://www.wrhc2018.com/pdf/Rural%20Primary%20Care%20Report.pdf, contains valuable insights for PHC in Africa. The recommendations that arose from this consultation were framed around increasing investments in and access to PHC, supporting PHC teams to take responsibility for the health and wellness of defined populations, and training and retaining appropriate healthcare professionals for rural PHC.

The conference itself culminated in the unanimous adoption of the Delhi Declaration, which was specifically intended to speak into the WHO plans (available at https://www.who.int/hrh/news/2018/delhi_declaration/en/). It called for special priority to be given to rural communities if nations are to achieve universal health coverage. In the spirit of the Alma Ata Declaration, it identified six priority areas that need to be addressed to achieve Health for All Rural People (HARP):

- achieving equity and access to good quality care for rural people
- a process of rural health impact assessment or ‘Rural Proofing for Health’ in terms of all policies related to health and healthcare
- health system development, particularly the development of multidisciplinary teams of health workers with skills to address the specific needs of rural and isolated communities, supported by digital health technologies
- developing and educating fit-for-purpose health professionals through specific rural training programmes
- realigning research to reverse the 90/10 research gap (90% of the world’s investment in health research addresses only 10% of the global health problems^1^) so that data from rural research can inform decision-making on rural health services
- investment in the infrastructure, services and economies of rural areas for a lasting impact on the well-being of their populations.

The Delhi Declaration arose out of the work of RuralWonca in lobbying to ensure that the needs of rural people, and those disadvantaged by rurality and the access issues that accompany it, are acknowledged by WHO and governments. This involvement goes back to 2002 when there was a joint WONCA-WHO consultation on HARP, from which various initiatives arose, including the ongoing series of world rural health conferences.^2^

Many different organisations took part in the Astana conference and provided their inputs leading up to the conference, including WONCA. The resulting Declaration of Astana (available at https://www.who.int/docs/default-source/primary-health/declaration/gcphc-declaration.pdf) is a political document – like the original Alma Ata Declaration – and suffers from the compromises that are inevitable in such documents, as various vested interests sought to have their concerns included; however, despite that, it is a significant document that can be used as an advocacy tool – all WHO member states, including all African countries, have supported it.

Access to care is emphasised, with the commitment that primary care services – promotive, preventive, curative, rehabilitative and palliative care – must be accessible to all people, which includes addressing issues of the cost of accessing care and shortages of healthcare workers, both absolute and relative. There is a specific recognition of the need to ensure that the PHC workforce is available to rural, remote and least developed areas of the world.

The declaration is short on detail, especially in terms of implementation. While WHO is developing an operational framework that may provide guidance for implementation, it is important that healthcare professionals involved in primary care use the document across countries in Africa to make a difference in and support the communities they serve.

What are the implications of this? There are, of course, many, but I offer a few personal thoughts in this regard. While there is a strong commitment to universal health coverage, too often this is conceptualised in terms of healthcare financing rather than focusing on access to healthcare for everyone regardless of geographical location, nationality, ethnicity, gender, ability or disability, or resources. Rural people are very often impoverished in the process of gaining access to ‘free healthcare’ – health must be delivered in and for communities, and they should be empowered to demand good quality care. Siloed approaches to care, including the funding of vertical and disease-focused programmes without significant accompanying health systems strengthening at primary care level, by governments or donors must be minimised and, where they exist, they must be managed within PHC teams. Appropriate training must be instituted so that healthcare professionals working in primary care, especially rural communities, have the range of skills needed to ensure that care is not, and is not seen to be, substandard. This includes ensuring that there is postgraduate family medicine training for physicians, and modernised, relevant education for associate clinicians (the so-called mid-level workers) and nurse practitioners. The regularisation and recognition of the status of community health workers and their meaningful inclusion into PHC teams are also critical.

We hope that the 50th Alma Ata anniversary in 2028 will be a celebration of PHC having gained its rightful place as the basis for health systems development across Africa.

## References

[1] Kilama WL The 10/90 gap in sub-Saharan Africa: Resolving inequities in health research. Acta Trop. 2009;112(Suppl 1):S8–S15. 10.1016/j.actatropica.2009.08.015 19695211

[2] Couper I, Strasser R, Rourke J, Wynn-Jones J Rural health activism over two decades: The Wonca Working Party on Rural Practice 1992–2012. Rural Remote Health. 2015;15(3):3245. 26219621

